# Mechanism-Based Pharmacokinetic and Pharmacodynamic Modeling

**DOI:** 10.3390/pharmaceutics18080961

**Published:** 2026-08-05

**Authors:** Artur Świerczek

**Affiliations:** Department of Pharmacokinetics and Physical Pharmacy, Faculty of Pharmacy, Jagiellonian University Medical College, Medyczna 9, 30-688 Kraków, Poland; artur.swierczek@uj.edu.pl

## 1. Introduction

Over the past two decades, pharmacokinetic and pharmacodynamic (PK/PD) modeling has moved from the empirical fitting of concentration time and concentration effect data toward a mechanistic discipline that represents the biology connecting drug exposure to response [1,2]. Mechanism-based models do not treat the exposure–response relationship as a black box. Instead, they embed the relevant physiology and pharmacology directly in their structure, including disposition across tissues, target binding and receptor dynamics, signal transduction, biomarker turnover, and disease progression [3]. This is what allows them to extrapolate: to untested doses and schedules, to new populations, and from preclinical systems to the clinic.

The maturation of the field has been driven by complementary paradigms. Physiologically based pharmacokinetic (PBPK) models resolve drug distribution tissue by tissue [4]; quantitative systems pharmacology (QSP) couples disposition to networks of interacting biological processes [5]; and population PK/PD models quantify variability and locate its clinically relevant sources [6]. The contributions to this Special Issue draw on all three, across oncology, anesthesia, cardiology, cannabinoid pharmacology, and generic drug development, and they address both small molecules and the systems-level behavior of endogenous proteins.

Mechanism-based modeling now sits at the center of model-informed drug development (MIDD) [7] and model-informed precision dosing (MIPD) [8]. Regulatory agencies increasingly accept modeling and simulation in support of dosing, biowaivers, and label claims, and they have issued dedicated guidance on PBPK [9] and population PK analyses [10], reflecting a broader shift toward model-based regulatory decision-making [11]. Against this background, this Special Issue of *Pharmaceutics* gathers rigorous applications of PK/PD modeling together with the methodological choices that make such models trustworthy.

## 2. Overview of Published Work

The eight contributions to this Special Issue are listed at the end; here I highlight six that together map the methodological breadth of the field.

One study exemplifies the reach of QSP. Hu and co-workers (Contribution 8) built a mechanistic QSP-PK/PD model of the sequence-dependent synergy between pemetrexed (PEM) and osimertinib (OSI) in non-small-cell lung cancer (NSCLC). With no relevant pharmacokinetic interaction between the agents, sequential dosing (pemetrexed followed by osimertinib) outperformed concurrent administration. Pemetrexed first inflicts damage and provokes a compensatory EGFR rebound that osimertinib then suppresses to drive apoptosis, and the simulations showed that osimertinib could follow pemetrexed by 48 h while retaining the benefit across polymorphisms and inter-individual variability.

Two further papers move from mechanism toward the bedside. Qian and Zhou (Contribution 4) developed a PBPK-PD model for the heart-rate effect of Δ9-tetrahydrocannabinol (THC) and its active metabolite 11-OH-THC, capturing 97% of observed heart rates within the 5th to 95th prediction percentiles and predicting tachycardia in about half of subjects at oral doses of 60 mg or inhaled doses of 15 mg. Chen and colleagues (Contribution 1) reported a population PK/PD analysis of remimazolam and its metabolite CNS 7054, linking exposure to the bispectral index (BIS) through an effect-compartment model and translating the result into a web-based dashboard for target-BIS dosing, a concrete instance of MIPD.

Regulatory biopharmaceutics is represented by Zarzoso-Foj and co-workers (Contribution 5), who applied physiologically based biopharmaceutics modeling (PBBM) to ibuprofen, identifying particle surface pH as the dissolution-controlling parameter and delimiting a safe space for virtual bioequivalence (BE). They also noted candidly where the virtual predictions overestimate, a useful reminder that such tools must be qualified for their regulatory context.

Two reviews frame the broader methodology. Świerczek, Batko, and Wyska (Contribution 2) survey the role of pharmacometrics in autoimmune disease, mapping the pharmacometric toolbox from PBPK, PK/PD and disease-progression modeling to population analysis, model-based meta-analysis, and QSP, together with the growing use of artificial intelligence (AI) and machine learning (ML), and showing how these methods sharpen dosing, improve safety, and anticipate patient-specific responses. Guan, Tu, and Yu (Contribution 6) provide a complementary, disease-focused review of practical PK/PD models in oncology, spanning compartmental and PBPKs, tumor-growth and disease-progression models, and the particular challenge of combination regimens.

The remaining contributions, a QSP-PBPK analysis of tafamidis and transthyretin (TTR) dynamics and a population model of propofol and remifentanil hemodynamics, are listed among the Contributions and further broaden the scope of the issue.

## 3. Future Perspectives

Several directions stand out. The first is the continued convergence of PBPK, QSP, and systems-pharmacology approaches into integrated, multi-scale models that link whole-body disposition to molecular and cellular pharmacodynamics, a convergence already visible across this issue. The second is a tighter coupling of mechanistic models with clinical decisions: MIPD needs models that are mechanistically sound, robust to inter-individual variability, and usable at the bedside, with an explicit context of use and quantified confidence [8].

Third, the expanding role of virtual BE and PBBM in regulatory science brings both promise and responsibility. Predictions that support biowaivers or labeling must be transparent, reproducible, and honestly weighed against their limits [9]. Finally, AI and ML will complement rather than replace mechanistic modeling, accelerating parameter estimation, model selection, and high-dimensional data analysis, while mechanistic structure preserves the interpretability and extrapolative power that data-driven methods lack [11].

I thank the authors for their contributions, the reviewers for their careful work, and the editorial staff of *Pharmaceutics* for their support. I hope that readers will find here both useful methodology and motivation to keep developing mechanism-based PK/PD modeling.

## Data Availability

No new data were created or analyzed in this study. Data sharing is not applicable to this article.
